# Characterization of Chelation and Absorption of Calcium by a *Mytilus edulis* Derived Osteogenic Peptide

**DOI:** 10.3389/fnut.2022.840638

**Published:** 2022-04-05

**Authors:** Zhe Xu, Shiying Han, Hui Chen, Zhixuan Zhu, Lingyu Han, Xiufang Dong, Ming Du, Tingting Li

**Affiliations:** ^1^Key Laboratory of Biotechnology and Bioresources Utilization, College of Life Sciences, Dalian Minzu University, Ministry of Education, Dalian, China; ^2^Key Laboratory of Marine Fishery Resources Exploitment and Utilization of Zhejiang Province, College of Food Science and Technology, Zhejiang University of Technology, Hangzhou, China; ^3^College of Marine Science and Biological Engineering, Qingdao University of Science and Technology, Qingdao, China; ^4^National Engineering Research Center of Seafood, Collaborative Innovation Center of Seafood Deep Processing, School of Food Science and Technology, Dalian Polytechnic University, Dalian, China

**Keywords:** *Mytilus edulis*, peptide, absorption, bone, calcium

## Abstract

In a previous study, the peptide LGKDQVRT, which was identified by enzymatic hydrolysis, released during the proteolysis of *Mytilus edulis*, had potential osteogenic activity. In this study, the octapeptide LGKDQVRT was able to spontaneously bind calcium in a 1:1 stoichiometric ratio, and the calcium-binding site likely involves calcium and amino acid VAL6 in the LGKDQVRT peptide to form a metal-donor to metal acceptor complex. The peptide LGKDQVRT has the activity of promoting the proliferation and differentiation of osteoblasts. The results of this study suggest that hydrolyzed peptides from *Mytilus edulis* protein can be used as a dietary supplement to improve calcium absorption and prevent osteoporosis.

## Introduction

Osteoporosis has developed into a global bone disease in recent years, and its mechanism of action is an increased risk of bone fragility and fracture, the degeneration of bone and bone tissue microstructure (1, 2). The disease poses an serious and social burden in both developed and developing countries. Scientists predict that in the next 20 years, there will be twice as many patients suffering from osteoporosis (3). Relevant studies predict that more than one-third of Chinese women after the age of 50 will suffer from osteoporosis, which will increase their fracture risk (4). It is a critical causes for metabolic bone diseases, such as osteoporosis, to calcium deficiency (5). Therefore, calcium supplementation is very important to prevent osteoporosis. However, some clinical trials have shown that commonly used calcium agents such as calcium citrate and calcium carbonate are not well absorbed by the body. These calcium agents will form precipitates in the gastrointestinal tract, leading to low bioavailability (6, 7). Although vitamin D can promote the absorption rate of calcium, high-dose vitamin D supplementation can increase the risk of fractures (8). Therefore, the improvement of calcium absorption efficiency is one of the effective means to prevent osteoporosis.

In healthy individuals, bone metabolism is a complex life process that continuously undergoes bone resorption and bone reconstruction. The biological cascade is that osteoclasts secrete proteases to digest the bone matrix in the old bone area, and form bone resorption lacuna. Then the osteoblasts move to the resorbed site, secrete the bone matrix, and the bone matrix is mineralized to form new bone (9). The dynamic balance between bone resorption and bone formation determines bone density and bone mass (10). When bone formation is less than bone resorption due to some pathological reasons, the balance is disrupted and people will suffer from osteoporosis. Bone resorption is regulated by osteoclasts and bone formation is regulated by osteoblasts. The dynamic balance between these two types of cells is the key to maintaining the stability of bone mass and minerals in the human body. In other words, osteoporosis can be prevented by promoting the proliferation and differentiation of osteoblasts, and the differentiation of osteoblasts is often accompanied by an increase in alkaline phosphatase (ALP) activity (10).

For the last few years, some food-based calcium supplements have received widespread attention. In particular, some food protein-derived peptides have been shown to be good carriers for calcium transport (11, 12). And some studies have shown that peptide-calcium chelates are more easily absorbed and utilized by mucosal cells in the intestine because of the chelating structure formed (13). The lack of calcium has been confirmed to be related to osteoporosis (14). Calcium-peptide chelate enhances calcium absorption and promotes bone tissue mineralization, thereby preventing osteoporosis (15). Many shellfish peptides have osteogenic activity (16, 17), among which *Mytilus edulis*-derived peptide LGKDQVRT was screened out in previous research results to have potential osteogenic activity (17).

The interaction between the peptide LGKDQVRT and calcium determines its chelating effect. The chelation effect and site of action were verified by techniques such as UV absorption spectroscopy, isothermal titration calorimetry, molecular docking, circular dichroism and MALDI-TOF MS. Furthermore, the peptide LGKDQVRT could not only promote calcium ion uptake under the Caco-2 model, but also promote osteoblast proliferation and its ALP activity. Therefore, the peptide LGKDQVRT has good promotion of calcium absorption and prevention of osteoporosis. This provides a theoretical basis for the development of functional foods for promoting calcium absorption and preventing osteoporosis.

## Materials and Methods

### Materials and Chemicals

Caco-2 cells and MC3T3-E1 cells were provided by the Cell Bank of the Chinese Academy of Sciences. The LGKDQVRT peptide with the ability to chelate calcium was screened and identified. And the LGKDQVRT peptide with a purity of 98.61% was prepared by Chinapeptides Biological Technology Co., Ltd through Fomc-protected amino acid synthesis method. The other analytical grade that all chemicals and reagents used in this study.

### Ultraviolet-Visible Absorption Spectroscopy

In order to verify whether there is a chelation reaction between calcium and peptide, this experiment used a spectrophotometer (UV-4802S, Uniko Instruments Co. Ltd, Shanghai, China) to measure the ultraviolet-visible spectra. As described previously (18), first dissolve the calcium-binding peptide in PBS buffer with a pH of 8.0 and a concentration of 20 μg/mL, and then add 0, 0.5, 1, 1 and 1 μL of 2 mol/L CaCl_2_ solution in sequence. Scanning and recording the results every 10 min at wavelengths ranging from 190 to 400 nm.

### Isothermal Titration Calorimetry

The affinity isothermal titration calorimetry (TA Instruments Ltd, New Castle, DE, USA) was used when conducting Isothermal Titration Calorimetry (ITC) experiments. It is 50 mM Tris/HCl buffer that the peptide and CaCl_2_ were mixed with, and filtered in a 0.22 μm membrane filter, then vacuum degassed for 10 min, and finally titrated. The CaCl_2_ solution was loaded into the syringe as a ligand, and LGKDQVRT was added to the reaction tank as the titrated substance. The syringe drips the ligand and interacts with the titrant in the sample cell to trigger the binding reaction. The CaCl_2_ solution was loaded into the syringe as a ligand, and LGKDQVRT was added to the reaction tank as the titrated substance. The syringe drips the ligand and interacts with the titrant in the sample cell to trigger the binding reaction. Every 200s, 2.0 μL of CaCl2 solution was titrated into 350 μL of LGKDQVRT solution for 25 automatic titrations, and the temperature was controlled at 25°C during the experiment. Titrate the CaCl_2_ solution as a blank titration experiment. Titrate the CaCl_2_ solution with Tris-HCl buffer as a blank titration experiment. For the stability of the solution and the accuracy of the experimental data, titrate three times for each experiment. The raw data obtained during the experiment were fitted using the “Independent Binding” mode of NanoAnalyze software, and the relevant thermodynamic parameters of the reaction between LGKDQVRT peptide and CaCl_2_ were calculated (19).

### Molecular Docking and Optimized Structure of the LGKDQVRT-Calcium Complex

When chelating peptides and calcium ions, use a four-step docking procedure. First, use the Discovery Studio 2017 of the molecular simulation software from Accelrys to create a new calcium ion model. In the second step, enter the peptide sequence into “Build and edit protein” Module, and then use the Protein Prepare Tool to optimize the bond length of each peptide and small ligand molecules (20). In the third step, calcium ions are used as ligands and the complete peptides are used as receptors. The ligands and receptors are used for molecular docking using the Dock Ligands (CDOCKER) (21) protocol tool in the Discovery Studio 2017 software. Finally, use the Receptor-Ligands-Interaction tool in the Discovery Studio 2017 software to analyze the molecular docking results, such as docking energy, distance and chelation type.

### Circular Dichroism

The Jasco J-810 Circular Dichroic Spectrometer produced by JASCO Japan Spectrometer was used to determine the secondary structure of LGKDQVRT peptide, LGKDQVRT-calcium complex and its simulated GI digestion. Each sample was scanned three times in the far ultraviolet region of 190–260 nm. The sample concentration is 1 mg/mL.

### MALDI-TOF MS Analysis

The MALDI-TOF method is widely used in the identification of peptide molecular weight and the quantification of relative content (22). Dispersing LGKDQVRT-calcium with a concentration of 0.5 mg/mL in 0.1% TFA buffer (water/methanol, 50/50, v/v), and TFA buffer as the matrix. Separately draw 1 μL of matrix and internal standard and mixed with the dry sample to obtain a mixture. Take 0.5 μL of the mixture and drop it on a stainless-steel target plate, then dry it and perform MALDI-TOF MS analysis. The mass spectrum was collected by a SmartBeam II laser with a parameter of 355 nm in reflection mode, showing a repetition rate of 200 Hz and an accelerating voltage of 20 kV. The number of laser shots in each experiment is 200. Data analysis was performed using FlexAnalysis. The mass accuracy was 50 ppm and the sample molecular peaks were compared with the peptide LGKDQVRT.

### MTT and ALP Activity Analysis

The proliferation effect of peptide LGKDQVRT on MC3T3-E1 cells was measured by MTT method (17). MC3T3-E1 cells were cultured in 96-well plates at 5,000 cells per well. After 24 h, treatment was performed with or without the addition of the peptide LGKDQVRT for 24 h. Then 10 μL of MTT at a concentration of 5 mg/mL was added. After incubation, replace the supernatant with 150 μL of DMSO solution. Finally, the assay was performed with a microplate reader (Infinite™ M200, TECAN, Switzerland) at a wavelength of 570 nm.

The effect of the peptide LGKDQVRT on the ALP activity of MC3T3-E1 was determined with reference to the kit instructions (Beyotime, Haimen, China) (23).

### Calcium Absorption Determination

Caco-2 cells were purchased from the Cell Resource Center of Shanghai Institute of Biological Sciences, Chinese Academy of Sciences (Shanghai, China). The formula of minimal essential medium (MEM) is 1% penicillin-streptomycin-neomycin antibiotic mixture and 20% fetal bovine serum. The Caco-2 cell monolayer model was established and studied according to the methods of previous studies (24, 25), the resistance value of the monolayer in this calcium transport experiment is >500 Ω/cm^2^. 0.5 mM peptide LGKDQVRT and 5 mM CaCl_2_ or 1 mM peptide LGKDQVRT and 5 mM CaCl_2_ were dissolved in 0.5 mL of Hank's balanced salt solution (HBSS) pH 7.4 and added to the upper layer, respectively. Cells are cultured in the medium, and then incubated in a constant temperature and humidity incubator with a temperature of 37°C and a humidity of 5% CO_2_ for 2 h.

At 30, 60, 90, and 120 min, draw sample to be tested from the basolateral side, and add the same volume of fresh HBSS buffer to keep the volume constant. Use atomic absorption spectrophotometer (Hitachi, Tokyo, Japan) to determine the calcium content in the sample to be tested.

### Statistical Analysis

Use the mean ± standard deviation to represent the experimental results. The significance of the experimental results was analyzed with SPSS 19.0 software, and Duncan (D)'s post-test statistical method was used in the analysis. *p* < 0.05 indicates that the results are significantly different.

## Results and Discussion

**Figure 1:**
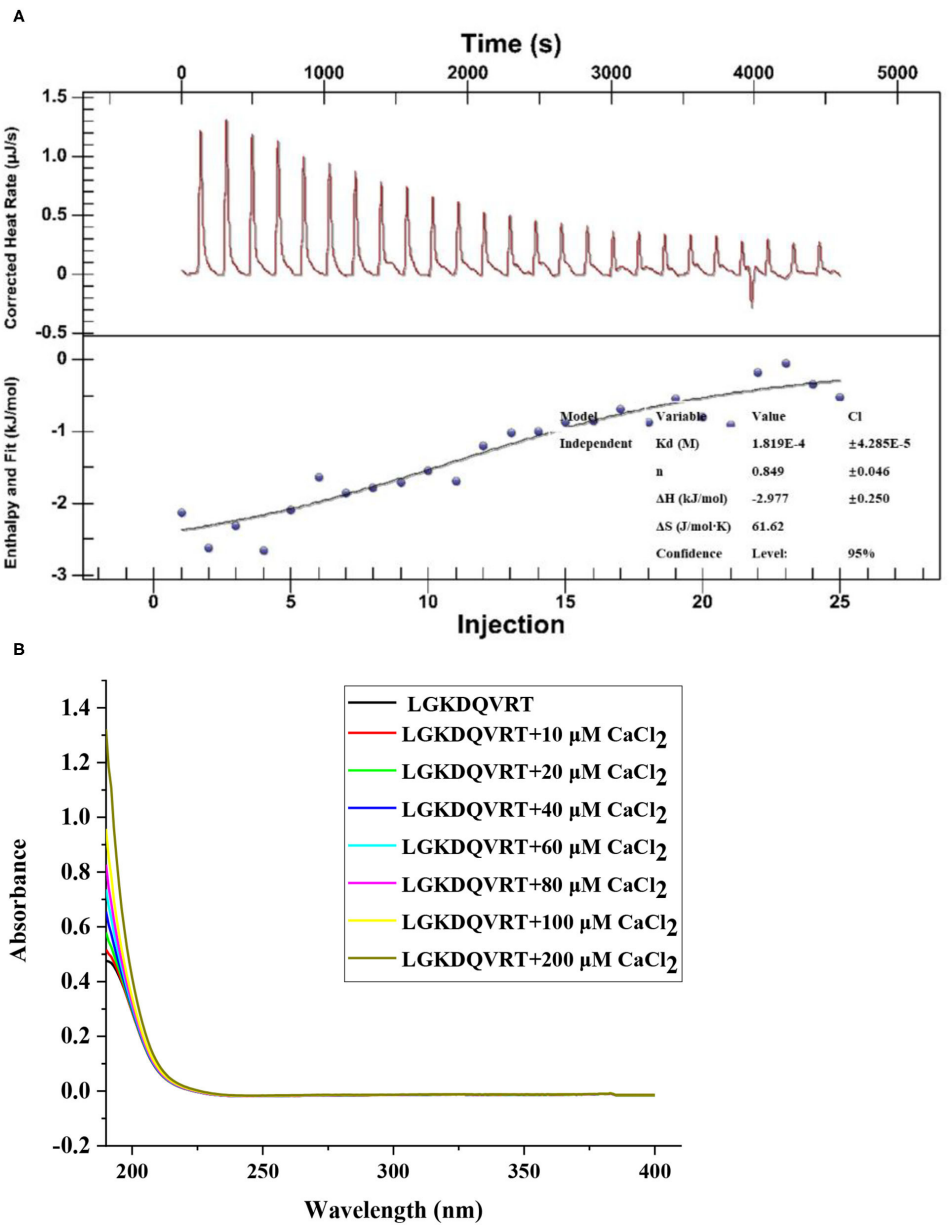
**(A)** The raw ITC data obtained from continuous injection of 2.0 μL CaCl_2_ solution to LGKDQVRT solution. Titration plot derived from the integrated heats of binding of the raw ITC data. **(B)** UV spectra of LGKDQVRT with different CaCl_2_ concentrations over the wavelength range from 190 to 400 nm.

The formation of LGKDQVRT-calcium complex was analyzed by ultraviolet-visible (UV) spectroscopy. As a result of the formation of a complex composed of metal ions and organicligands, it will cause the existing absorption peaks to move or disappear or to form new absorption peaks (26). According to Figure 1A, when Ca^2+^ was added to LGKDQVRT, the absorption intensity at a wavelength of 190 nm became higher. This can be seen as amide bonds, carboxyl groups and carbonyl groups in peptides. And there was another phenomenon, due to the addition of Ca^2+^, the absorption peak shifted from 0.4757 to 1.3219, which indicated that more calcium reacts with LGKDQVRT and different electronic transitions occur (26). In addition, the change of absorption peak intensity may also be caused by the oxygen atom of the carbonyl group and nitrogen of the amino group in the peptide bond in the peptide LGKDQVRT (27). The experimental results show that after LGKDQVRT is combined with Ca^2+^, the chromophore groups (–COOH, C=O) and auxochromes (–NH_2_, –OH) of the peptide have changed (18). This finding shown that the LGKDQVRT chelates calcium to form the LGKDQVRT–calcium complex.

ITC technology is a classic method for measuring the interaction between biomolecules. It can measure the change of heat during the reaction and provide accurate thermodynamic data (28). As shown in Figure 1B, when calcium ions titrated LGKDQVRT, an exothermic combined isotherm was produced, and the corresponding n value is close to the experimental conditions of 1. Under the conditions of pH 6.4 and temperature 37°C, a negative enthalpy (ΔH = −2.977 ± 0.250 kJ/mol) was produced during the experiment, indicating that the reaction proceeded spontaneously. Due to the lower entropy (ΔS = 61.62 J/mol·K), it shows that hydrophobic interaction and conformational freedom have less effect on the formation of complexes.

**Figure 2:**
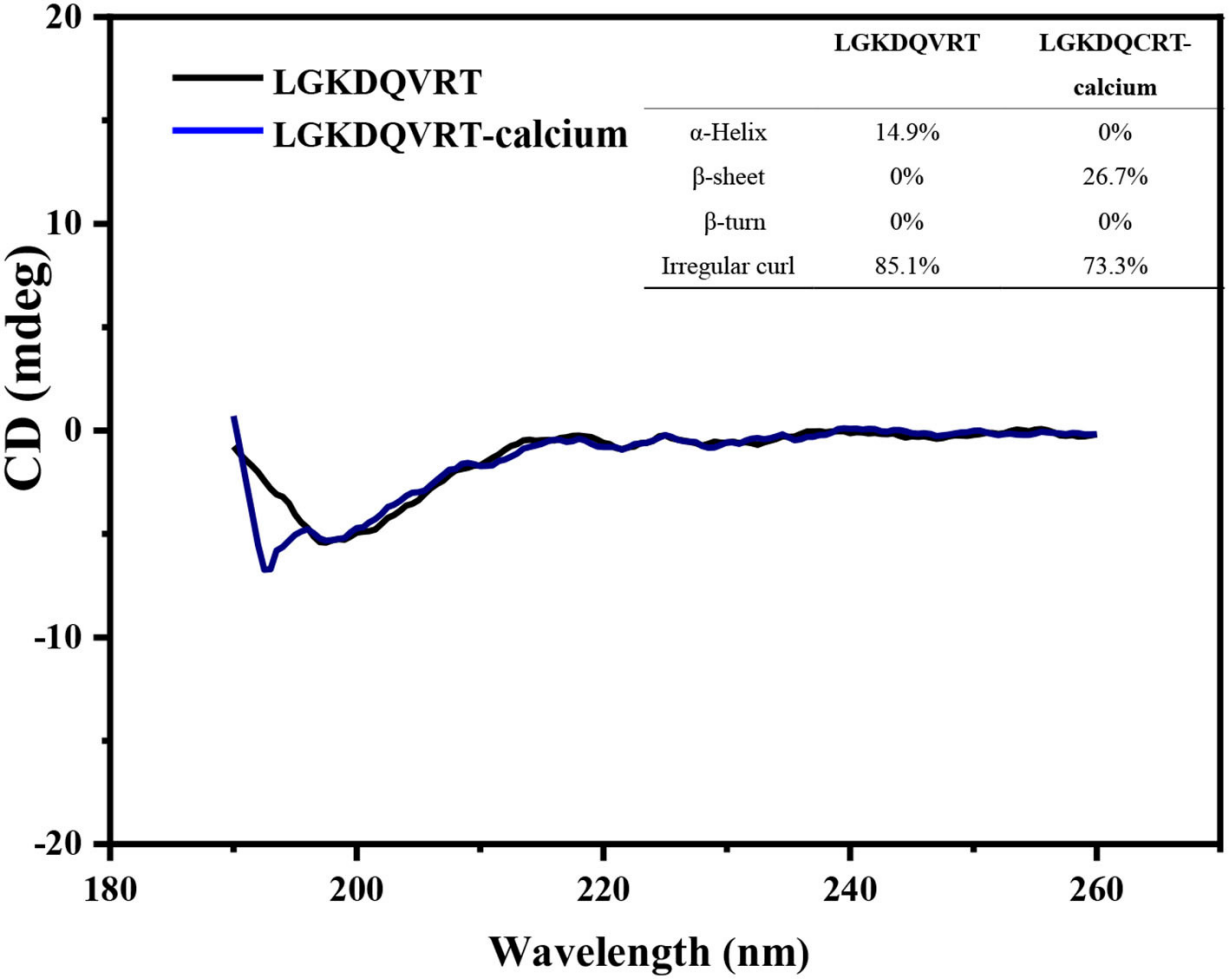
Far-UV circular dichroism spectroscopy of the LGKDQVRT and LGKDQVRT-calcium complex over the range of 190–260 nm, insets show their secondary structure relative content of α-helix, β-sheet, β-turn and random coil.

The chelation behavior of peptides and metals will change the secondary structure of the peptide, such as α-Helix, β-turn and so on. CD spectroscopy has the advantages of rapid, simple and accurate determination of protein secondary structure, and it is the most widely used tool for studying protein secondary structure (29). The CD spectrum obtained from the experimental results can evaluate the influence of Ca^2+^ on the secondary structure of LGKDQVRT peptide, as shown in Figure 2. When the LGKDQVRT peptide is in water, there is a large negative CD peak at about 197 nm, which indicates that the secondary structure of the peptide is a typical random coil conformation (30). When the LGKDQVRT peptide is combined with calcium ions, there is a much smaller negative CD peak at ~197 nm, which indicates that the secondary structure of the random coil is significantly reduced compared with the LGKDQVRT peptide molecule alone (*p* < 0.05). Because calcium ions bind to the affinity site of LGKDQVRT peptide to form LGKDQVRT-calcium nanocomposite, the α-Helix and irregular curl conformations are reduced, while the β-turn conformation is increased. This makes the protein structure more orderly and compact revealing that LGKDQVRT peptide fold and form a porous network structure after binding calcium ions, which is consistent with the results of Cui et al. (31).

**Figure 3:**
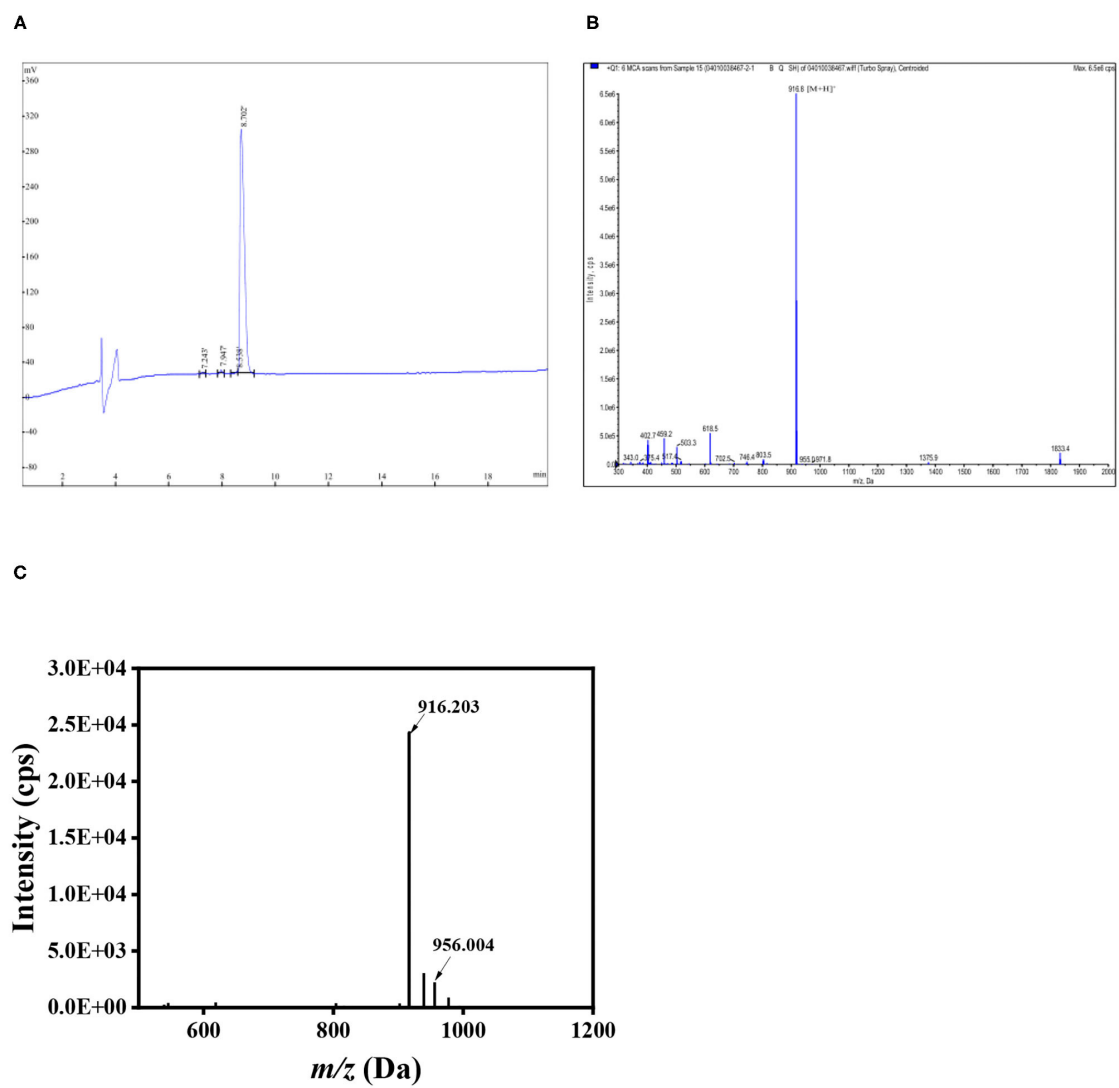
Peptide LGKDQVRT identified by HPLC and Mass spectrum. **(A)** HPLC of LGKDQVRT. **(B)** Mass spectrum at the m/z of 916.8 Da for LGKDQVRT. **(C)** LGKDQVRT-calcium complex identified using MALDI-TOF MS.

The peptides LGKDQVRT and LGKDQVRT-calcium were identified by scanning using matrix-assisted laser desorption ionization-time of flight mass spectromentry (MALDI-TOF MS) (32). The m/z of LGKDQVRT was determined by MALDI-TOF to be 916.203, while its molecular weight was 916.8, proving that the instrument is accurate. In Figure 3, there is shown a peak at m/z 956.004 which differs from the measured 916.203 by 39.801 close to the molecular weight of calcium. Thus, the formation of LGKDQVRT–calcium complex is consistent with the results presented in Figure 1.

**Figure 4:**
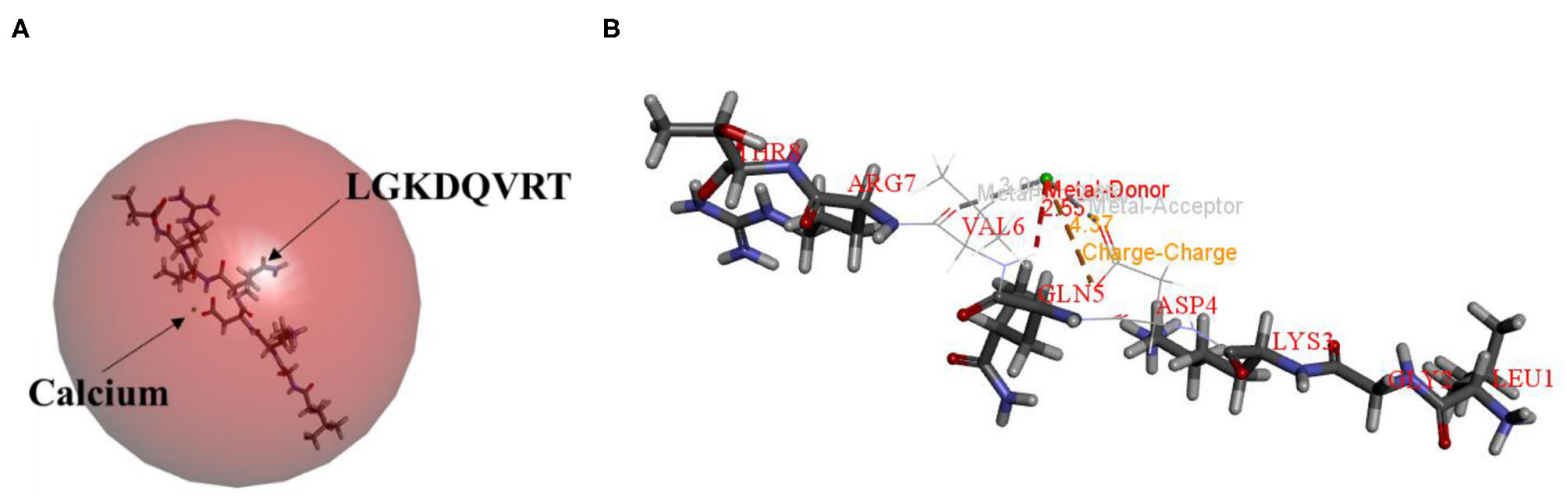
Molecular docking and optimized structure of the LGKDQVRT-calcium complex. **(A)** 3D structure of LGKDQVRT-calcium complex as a surface image, generated by the surface menu of Discovery Studio 2017 software. **(B)** Interaction among ASP4, GLN5, and VAL6 in LGKDQVRT with calcium.

Molecular docking technology is mainly used to study the interaction between molecules (such as ligands and receptors) (17, 33, 34). Therefore, the molecular docking technique was used to analysis the possible calcium-chelating mechanism in LGKDQVRT. As shown in Figure 4A, calcium was docked with LGKDQVRT as the center. Calcium and the amino acid VAL6 in the peptide form metal donor to metal acceptor complexes in Figure 4B, the chelation of calcium ions with metal acceptor for ASP and GLN of the LGKDQVRT peptide. As shown in Figure 4B, the docking type is a metal acceptor, which shown the interaction between carbonyl groups and calcium ions. This result is consistent with that of a previous report shown that calcium should form complexes with negatively charged carboxylate groups (35).

**Figure 5:**
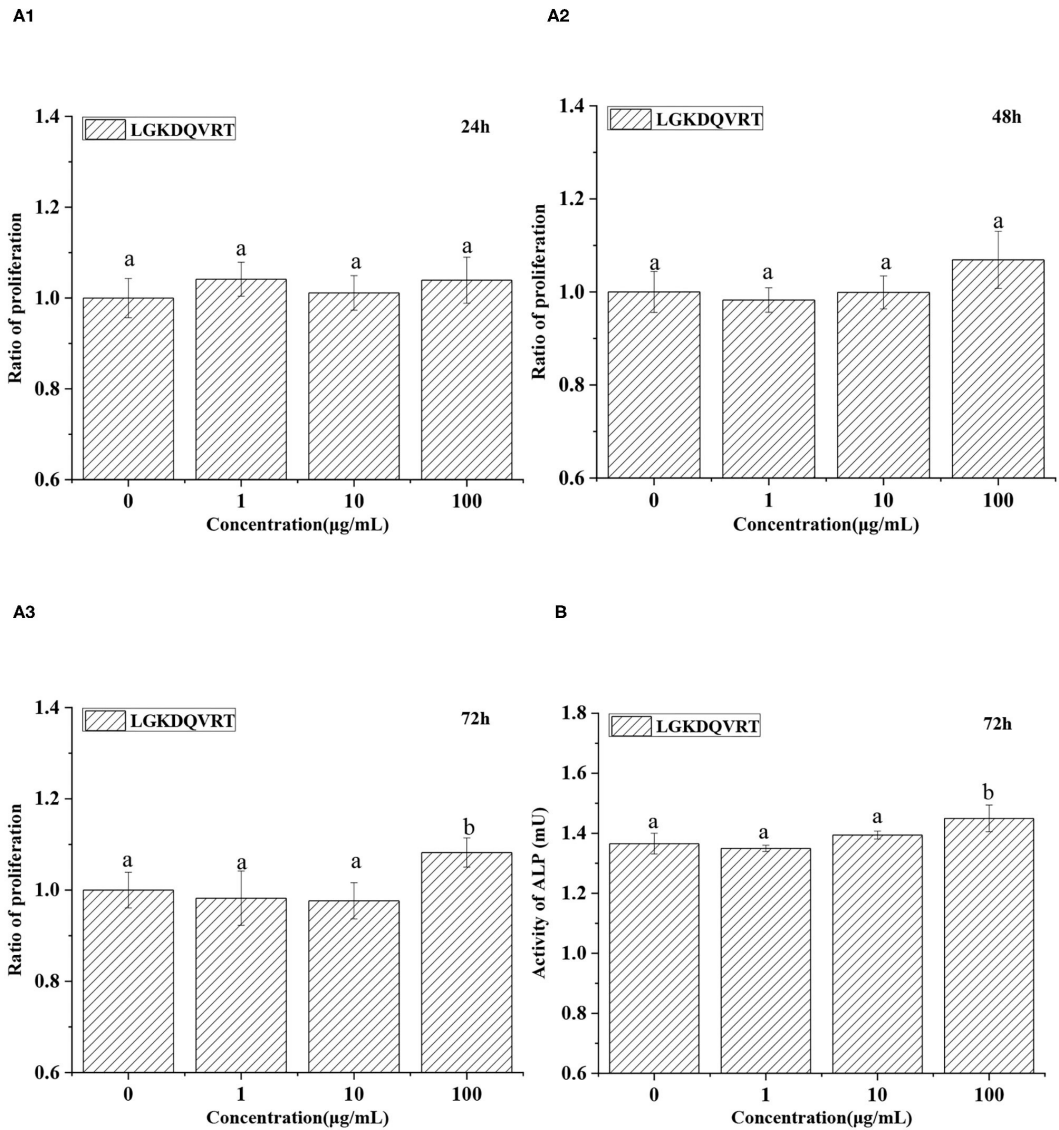
**(A)** Effects of 1, 10, and 100 μg/mL peptide LGKDQVRT for osteoblasts at 24, 48, and 72 h. **(B)** Effects of 100 μg/mL peptide LGKDQVRT for osteoblasts on ALP activity at 72 h. All statistical tests (*n* = 6) were conducted, and values designated by different letters are considered significantly different (*p* < 0.05).

Osteogenic peptide helps the proliferation and differentiation of osteoblasts (36, 37). Osteoblasts were cultured for 24, 48, and 72 h in medium with 1, 10, and 100 μg/mL LGKDQVRT, respectively. After treatment with LGKDQVRT at a concentration of 100 μg/mL for 24 h, 48 h and 72 h, the experimental results showed that the cell growth increased by 3.93%, 6.89% and 8.22% (*p* < 0.05) (Figure 5A). There were significant differences in the measured cell proliferation between the control group without peptide and the experimental group with 1, 10, and 100 μg/mL LGKDQVRT. In addition, the ALP activity of the control group was 1.3653 ± 0.0347 mU, and the ALP activity of adding 100 μg/mL LGKDQVRT was 1.4494 ± 0.0449 mU, an increase of 6.16% (Figure 5B). This is similarly to the previous results of peptides with osteogenic activity (38).

**Figure 6:**
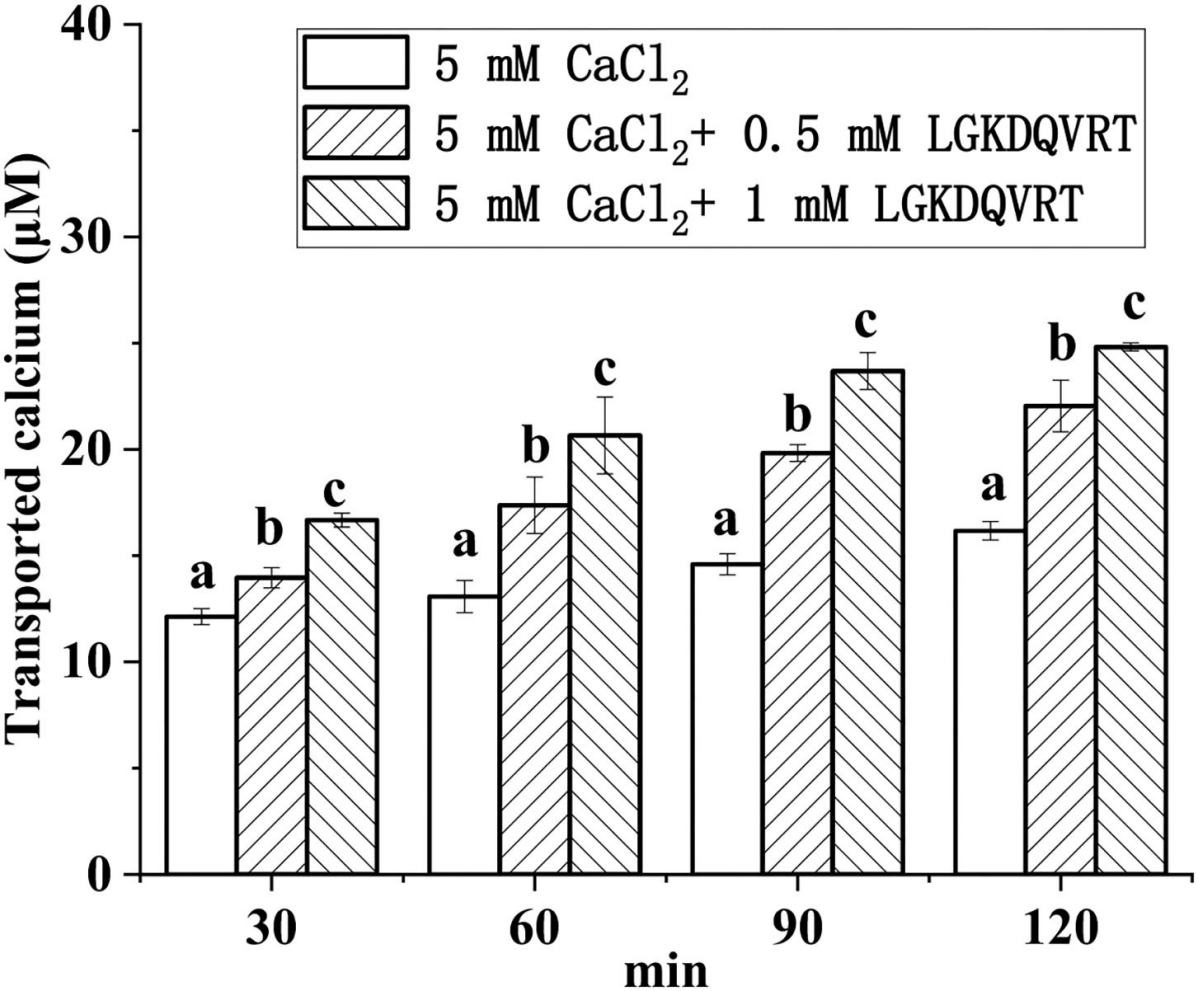
Calcium transport studies. Different concentrations of the LGKDQVRT (0.5 and 1 mM) or 5 mM CaCl_2_ in 0.5 mL of Hank's balanced salt solution (HBSS, pH 7.4) were added to the apical side, and incubated at 37°C for 2 h. Aliquots were extracted from the basolateral side at 30, 60, 90, and 120 min, and an equal volume of HBSS buffer was added to the basolateral side to keep the volume constant. Calcium contents were determined by using an atomic absorption spectrophotometer. Different letters indicate significant difference among the groups at the given time point (*p* < 0.05).

Caco-2 cell monolayer model was used to monitor the absorption of calcium ions triggered by LGKDQVRT *in vitro*. Different concentrations of CaCl_2_ and peptide LGKDQVRT were added to the upper side of the culture plate, and then samples were taken at four time points of 30, 60, 90, and 120 min, respectively. As shown in Figure 6, as the concentration of peptide LGKDQVRT increased, its effect on calcium absorption became more pronounced. The peptide LGKDQVRT promotes calcium absorption and transport. In conclusion, the peptide LGKDQVRT was beneficial for calcium uptake in Caco-2 cell monolayers.The peptide LGKDQVRT was consistent with the previously reported results of the peptide IEELEEELEAER, showing both relatively stable calcium absorption and good osteogenic activity (39, 40). Therefore, the peptide LGKDQVRT can not only promote the proliferation of osteoblasts but also promote calcium transport and absorption in the Caco-2 model *in vitro*.

## Conclusions

In my research, a new type of octapeptide LGKDQVRT derived from *Mytilus edulis* mimic digestive hydrolysate can be chelated with calcium at a stoichiometric ratio of 1:1. The binding of peptide LGKDQVRT to calcium is an exothermic process. Calcium and the amino acid VAL6 in the peptide form metal donor to metal acceptor complexes. Octopeptide LGKDQVRT can not only promote calcium absorption and increase calcium bioavailability in the Caco-2 model, but also promote osteoblast proliferation and differentiation to prevent osteoporosis. According to the above research results, it is feasible to produce natural peptide chelated calcium as a functional nutritional food for preventing osteoporosis.

## Data Availability Statement

The original contributions presented in the study are included in the article/supplementary material, further inquiries can be directed to the corresponding author/s.

## Author Contributions

TL and ZX provided the project administration and funding acquisition. ZX designed the research and wrote the manuscript. SH and ZZ executed the experiments and analyzed the data. HC, LH, XD, and MD reviewed and edited this manuscript. All authors have read and agreed to the published version of the manuscript.

## Funding

This work was supported by the National Key R&D Program of China (2018YFD0400601) and the Zhejiang Provincial Key Laboratory of efficient development and utilization of deep blue fishery resources projects to be funded by the open fund (SL2021004).

## Conflict of Interest

The authors declare that the research was conducted in the absence of any commercial or financial relationships that could be construed as a potential conflict of interest.

## Publisher's Note

All claims expressed in this article are solely those of the authors and do not necessarily represent those of their affiliated organizations, or those of the publisher, the editors and the reviewers. Any product that may be evaluated in this article, or claim that may be made by its manufacturer, is not guaranteed or endorsed by the publisher.
